# Utilisation of outpatient physiotherapy in patients following total knee arthroplasty – a systematic review

**DOI:** 10.1186/s12891-021-04600-2

**Published:** 2021-08-18

**Authors:** Hannes Jacobs, Gesine H. Seeber, Katharina Allers, Falk Hoffmann

**Affiliations:** 1 Department of Health Services Research, Carl von Ossietzky University Oldenburg, Ammerländer Heerstr. 114-118, 26129 Oldenburg, Germany; 2 University Hospital for Orthopaedics and Trauma Surgery Pius-Hospital, Medical Campus University of Oldenburg, Oldenburg, Germany; 3 Department of Orthopedics, University of Groningen, University Medical Center Groningen, Groningen, The Netherlands

**Keywords:** Physical therapy, Rehabilitation, Osteoarthritis, Health services research, Arthroplasty

## Abstract

**Objective:**

Data on the utilisation of outpatient physiotherapy (PT) in patients following total knee arthroplasty (TKA) are scarce, and available studies have not been systematically synthesised. This study aims to summarise the existing literature on outpatient PT following TKA as well as to identify factors associated with its use.

**Methods:**

A systematic literature search in MEDLINE (via PubMed), CINAHL, Scopus and PEDro was conducted in July 2020 without language restrictions. Two authors independently selected studies, extracted data and assessed study quality. The primary outcome was the proportion being treated with at least one session of outpatient PT (land- or water-based treatments supervised/provided by a qualified physiotherapist) during any defined period within 12 months following TKA. Furthermore, predictors for the use of PT were assessed. Studies including only revision surgeries or bilateral TKA were excluded.

**Results:**

After screening 1934 titles/abstracts and 56 full text articles, 5 studies were included. Proportions of PT utilisation ranged from 16.7 to 84.5%. There were large variations in the time periods after hospital discharge (4 weeks to 12 months) and in the reporting of PT definitions. Female sex was associated with higher PT utilisation, and compared to patients after total hip arthroplasty, utilisation was higher among those following TKA.

**Conclusion:**

Despite using a broad search strategy, we found only 5 studies assessing the utilisation of PT after hospital discharge in patients with TKA. These studies showed large heterogeneity in PT utilisation, assessed time periods and PT definitions. Clearly, more studies from different countries with uniform PT definitions are needed to address this relevant public health question.

**Supplementary Information:**

The online version contains supplementary material available at 10.1186/s12891-021-04600-2.

## Background

Osteoarthritis (OA) is the most common joint disorder and a leading cause of disability in older adults [1, 2]. It is a progressive condition associated with pain, movement restrictions and, as a result, diminished quality of life [3, 4]. Treatment of OA aims to manage symptoms and increase mobility, but at present there is no cure for this syndrome. For severe OA that no longer responds to medication and physiotherapy (PT) including exercise, clinical guidelines recommend total knee arthroplasty (TKA) as a cost-effective intervention [5–7]. This is one of the most frequently performed surgeries in industrialised countries [8, 9], and OA is by far the main indication for this procedure [8, 10, 11]. Due to demographic change and growing obese populations in high-income countries, rising OA prevalence as well as an increasing number of TKAs is to be expected [12–14].

TKAs are highly successful in restoring joint function and relieving pain, and they are associated with high patient satisfaction [15]. However, despite good results following surgery, a considerable proportion of patients undergoing TKA experience an unfavourable outcome. In a systematic review of prospective studies in unselected patients, 10 to 34% reported chronic pain between 3 months and 5 years after surgery [16]. Jones et al. observed 20% of patients with unfavourable pain outcomes at 6 months in a high-quality study in multiple centres with low loss to follow-up [17]. However, the exact percentage of patients with chronic pain and/or decreased function is unclear and varies across studies [16].

Physiotherapy, aiming to improve muscle strength, neuromotor control and range of joint motion [18] can be an effective treatment after TKA to help prevent unfavourable outcomes [19, 20]. Patients can receive various health care services that might also include PT in settings ranging from inpatient rehabilitation facilities to outpatient private practices and telerehabilitation. In (inpatient) acute care settings, PT is a routine component of postoperative management [19, 21] and can help to achieve a higher range of motion (ROM), reduce pain, and decrease the length of hospital stay [22]. Evaluating post-discharge rehabilitation in a systematic review, Artz et al. found improved physical function and pain at 3–4 months after surgery. Considering only higher quality studies, the benefit was apparent even up to 6 months [20]. However, although a Delphi study from Canada and the United States [23] published in 2014 already recommended PT for post-acute or post-discharge rehabilitation after TKA, rehabilitation programs currently vary greatly, and no clear prevailing evidence-based practice guidelines are available [24, 25]. Westby et al. call for supervised rehabilitation interventions provided by trained health professionals early after discharge from the acute care setting. In addition, regular follow-up visits with medical professionals (including PTs) in the late post-surgical phase (3–12 months) or even in the first 2 years following TKA is desirable to optimise patient outcomes [23].

Utilisation of PT prior to TKA was assessed by several studies. These studies found that 44 to 73% received PT prior to TKA [26–30] and that female sex [26–30], younger age [30] and higher education [30] were associated with an increased use of PT. After TKA, most studies were conducted in acute care settings, where PT is routinely used [19]. These studies often focus on differences in type, number and duration of PT and found that rehabilitation practice varies widely [19, 20]. However, data on the utilisation of outpatient PT in patients following TKA after acute care or inpatient rehabilitation are scarce, and the available studies have not been systematically synthesised (e.g. in terms of prevalence and factors associated with PT use).

Therefore, this study aims to summarise the existing literature regarding the proportion of patients receiving outpatient PT following TKA as well as to identify factors associated with its use.

## Materials and methods

A protocol for this systematic review was registered with PROSPERO (CRD42020197301). Furthermore, we followed the Preferred Reporting Items for Systematic Reviews and Meta-Analyses (PRISMA) statement [31].

### Data sources and search

The literature search was performed in the databases MEDLINE (via PubMed), CINAHL, Scopus and PEDro (see Additional file 1 for search strategy). We searched the electronic databases from inception to 09 July 2020. Additionally, we scanned the reference lists of all included studies.

### Eligibility criteria

We defined study eligibility criteria using the CoCoPop (condition, context, and population) approach for reviews assessing prevalence and incidence data [32, 33].

### Condition

We included studies reporting on proportions of patients receiving at least one session of outpatient PT during any defined period (e.g. 6 weeks, 6 months or 12 months) in the early phase (≤12 months) following TKA. We defined PT as land- or water-based treatments supervised or provided by a qualified physiotherapist regardless of content, duration, frequency or intensity. Therefore, studies assessing other forms of therapy not advised or provided by a qualified physiotherapist were not considered. Furthermore, studies reporting only mean amounts of treatments without proportions were excluded.

### Context

We included studies providing information on the use of PT during the rehabilitative period after hospital discharge following TKA surgery to the outpatient, community, or home setting. There was no exclusion based on special settings (e.g. nursing homes), but we excluded studies reporting on inpatient PT such as in acute hospitals or inpatient rehabilitation facilities.

### Population

Studies reporting on patients who underwent unilateral or bilateral TKA were included. Studies including only revision surgeries or bilateral TKA were not considered eligible. There were no restrictions regarding the type of hospital (community (general), teaching, rural, urban, or federal government hospital) or the number of hospitals from which participants were recruited. There were no limitations regarding age, sex or functional status of included patients, but we excluded studies that were limited to specific diagnoses beyond osteoarthritis (e.g. rheumatoid arthritis).

We included published observational and interventional studies. To be included, interventional studies had to fulfil one of the following conditions: [1] If information on outpatient PT was reported at follow up for patients recruited during index hospitalisation for TKA and placed in the control group (treatment as usual/usual care), we included the control arm [2]. If patients were recruited in any period within the first 12 months following TKA and the utilisation of outpatient PT after TKA was reported at baseline, both the intervention group and the control group were included. We excluded PhD theses and studies or the respective study arm in interventional studies with a sample size < 100 to ensure robust results. No other limitations, such as language, year or location of publication, were applied.

### Study selection and data extraction

Results from the literature search were exported into an EndNote (Version X9, Clarivate, Philadelphia, PA, USA) library, and duplicates were removed. Two of the authors independently screened articles based on title and abstract to determine inclusion or exclusion. Disagreement was resolved by discussion or by a third reviewer. Subsequently, the full text of all articles meeting the criteria was assessed by the two reviewers, and if necessary, any discrepancy was again solved by discussion or by a third reviewer.

We abstracted data on study characteristics (country, data source, year of data, sample size, number of hospitals, eligibility criteria), patient characteristics (mean age, sex) and outcome results (definition of PT, proportion of being treated with PT, period after hospital discharge, and factors affecting / predictors for the use of PT). Data extraction was performed by one reviewer and verified by a second. Discrepancies were resolved by discussion or by a third reviewer.

### Quality assessment

To assess the quality of the included studies, we chose the Joanna Briggs Institute’s (JBI) critical appraisal checklist for studies reporting prevalence data. It includes nine items [32] and is very flexible across different study designs [34]. Two reviewers independently assessed the quality of included studies. Any disagreement was resolved by discussion or a third reviewer. The assessed study quality had no impact on the inclusion or exclusion of studies.

### Data synthesis

We analysed the results using a narrative synthesis. Due to the expected heterogeneity between studies, a meta-analysis was not planned. Differences in PT utilisation by age, sex, socioeconomic status (SES) or physical function were analysed to the extent they were reported (irrespective of whether proportions were provided for these subgroups or whether variables were included in regression models). Additionally, reported information on other factors influencing the use of PT was assessed.

Beyond that, we aimed to compare PT use data from studies which also included patients following primary total hip arthroplasty (THA).

## Results

### Literature search

**Fig. 1:**
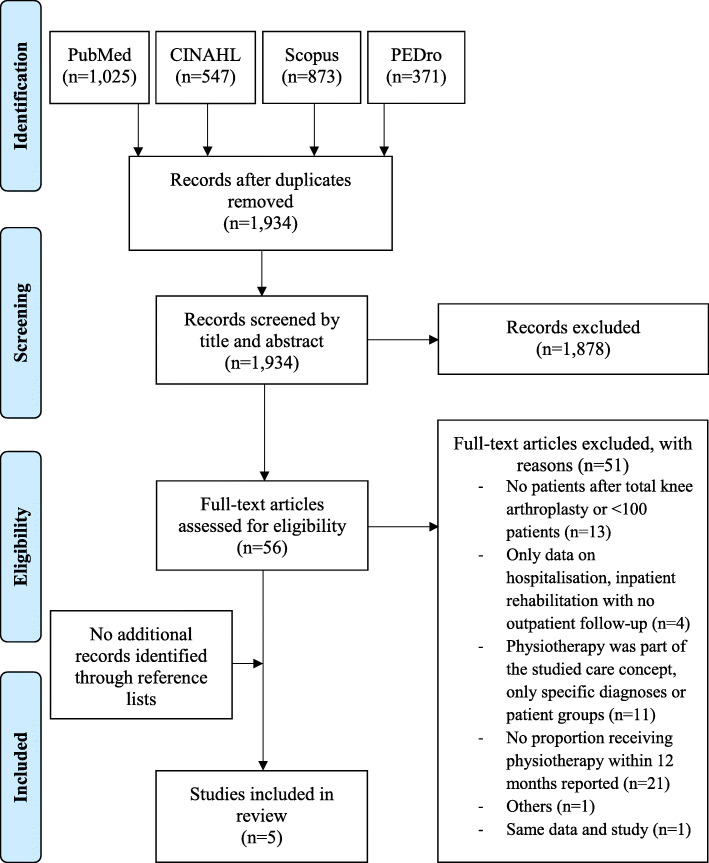
Flowchart of the literature search

The screening of 1934 titles/abstracts and 56 full text articles identified six studies fulfilling the inclusion criteria [35–40]. As two articles [37, 40] referred to the same study and data, we included only one of them [37]. Therefore, five full texts were finally included in the review [35–39] (Fig. 1). Four studies were reported in English and one in Danish.

### Study characteristics

Baseline characteristics of the five included studies are shown in Table 1. Two studies were conducted in the United Kingdom [36, 38] and one each in Denmark [35], Australia [37], and the United States [39]. Four studies used a longitudinal cohort design (two prospective and two retrospective), and one study used a randomised controlled trial design.

**Table 1:** Baseline characteristics of the studies included

First author, year	Country	Data Source	Year of data	Sample size	Number of hospitals	Inclusion/exclusion criteria	Mean age (% females)
Andersen 2009 [35]	Denmark	Linkage of National patient register (LPR) and Health Insurance Register (SSR)	2006	102	3	Inclusion: - All patients undergoing acute or elective primary TKA surgery regardless of age Exclusion: - Length of stay in hospital ≥20 days	70.2 (62.0%)
Hamilton 2019 [36]	United Kingdom	Self-reported questionnaire	01/2013 to 09/2015	1374 (THA = 1395)	1	Inclusion: - All patients undergoing unilateral primary TKA and THA regardless of age Exclusion: - Not reported	69.7 (56.6%)
Han 2015 [37]	Australia	Study-specific diary during the 6-week, post-discharge period	09/2009 to 10/2012	196	10	Inclusion: - All patients between 45 and 75 years undergoing elective/planned unilateral or bilateral primary TKA - Eligibility for being discharged into home environment Exclusion: - Previous unicompartmental arthroplasty/surgery of the same knee within the last 12 months - Comorbidity precluding exercise at 50–60% maximum heart rate, rheumatoid arthritis or major neurologic conditions	65.4 (53.0%)
Smith 2020 [38]	United Kingdom	Linkage of self-reported questionnaire and NJR	12 months between 2009 and 2010	20,260 (THA = 17,338)	Not reported	Inclusion: - All patients undergoing primary TKA or THA performed in England Exclusion: - Patients not registered in NJR and not part of NJR longitudinal PROMs programme	69.1^a^ (56.3%)^a^
Warren 2018 [39]	United States	Linkage of Centres from Medicare & Medicaid Services (CMS) and Health Resources and Services Administration’s (HRSA) Area Health Resource File (AHRF)	07/2010 to 06/2011	3482	2664	Inclusion: -All patients undergoing TKA (DRG codes 469, 470 and surgical procedure code 81.54) Exclusion: - Patients discharged to hospice, long-term care, or a psychiatric hospital or transferred to another acute care hospital - Patients who left against medical advice	71.3 (59.2%)

*Abbreviations*: *TKA* Total knee arthroplasty, *THA* Total hip arthroplasty, *DRG* Diagnosis-related group, *NJR* National Joint Registry for England, *PROMs* Patient Reported Outcome Measures

^a^Calculated from data given in the publication

The studies were published between 2009 and 2020, and the data were generated between 2006 and 2015. Use of PT was assessed in a variety of ways, with studies obtaining utilisation data from self-reported questionnaires, study-specific diaries, claims data, or registries. The sample size ranged from 102 to 20,260, and the number of hospitals in which participants underwent surgery ranged from 1 to 2664. One study did not report on the number of hospitals.

All studies reported data on patients’ age and sex. Females were in the majority in all five studies, with their proportion varying from 53 to 62%. Patients’ mean age ranged from 65 to 71 years.

### Methodological quality of included studies

The quality assessment for each study is presented in Table 2. Overall, in four of the five studies, the sample frame was appropriate for addressing the target population. In two studies, the sample size was inadequate. Another two studies did not conduct data analysis with sufficient coverage of the identified sample. Three of the five included studies identified the utilisation of PT with valid methods.

**Table 2:** Summary of quality assessment

First author, year	1	2	3	4	5	6	7	8	9
Andersen 2009 [35]	Yes	Yes	No	No	Yes	Yes	Yes	No	Yes
Hamilton 2019 [36]	Yes	Yes	Yes	Yes	Yes	No	Yes	Yes	Yes
Han 2015 [37]	No	Yes	No	Yes	No	Yes	Yes	Yes	Yes
Smith 2020 [38]	Yes	Yes	Yes	Yes	Yes	No	Yes	Yes	Yes
Warren 2018 [39]	Yes	Yes	Yes	Yes	No	Yes	Yes	Yes	N/A

Quality appraisal criteria [32]:

1) Was the sample frame appropriate to address the target population?

2) Were the study participants sampled in an appropriate way?

3) Was the sample size adequate?

4) Were the study subjects and the setting described in detail?

5) Was the data analysis conducted with sufficient coverage of the identified sample?

6) Were valid methods used for the identification of the condition?

7) Was the condition measured in a standard, reliable way for all participants?

8) Was there appropriate statistical analysis?

9) Was the response rate adequate, and if not, was the low response rate managed appropriately?

*Abbreviations*: *N/A* Not applicable

### Utilisation of outpatient PT following TKA

The definition of PT varied between studies and is presented in Table 3. One study defined PT as the proportion of patients visiting a physiotherapist (data extracted from a health insurance register), [35] and one study as provision (yes/no) of PT in a self-reported questionnaire [38]. A third study defined PT as billing codes used only by physical therapists [39]. Two studies did not report any definition of PT [36, 37].

**Table 3:** Results of the studies included

First author, year	Country (sample size)	Definition of PT	Proportion being treated with PT (period after hospital discharge)	Influence of age, sex, SES and physical function on the use of PT	Other factors influencing the use of PT
Andersen 2009 [35]	Denmark (*n* = 102)	Proportion of patients visiting a physiotherapist	16.7% (3 months)	Not reported	- Type of hospital ward: normal ward (16.7 and 13.8%) vs. fast track ward (6.7%)
Hamilton 2019 [36]	United Kingdom (*n* = 1374)	Not reported	48.2% (6 months) (THA: 35.3%)	- Age: OR: 1.04 (per year age decrease); 95% CI 1.02–1.05 - Sex: 50.1%^a^ (females) vs. 45.6%^a^ (males) and OR: 1.26 (for females): 95% CI 1.01–1.58^a^	Not reported
Han 2015 [37]	Australia (*n* = 196)	Not reported	84.5% (6 weeks)	Not reported	Not reported
Smith 2020 [38]	United Kingdom (*n* = 20,260)	Provision (yes/no and number of sessions) of PT as self-reported on questionnaires	79.0% (12 months) (THA: 53.0%)	- Sex: females (80.4%^a^) vs. males (77.7%^a^)	- Ethnicity: non-whites (85.4%^a^) vs. whites (79.2%^a^) - Living arrangements: family or spouse (80.8%^a^) vs. alone (78.1%^a^) vs. nursing home/hospital (78.9%^a^) vs. other (73.3%^a^) - ASA grade: fit and healthy (81.0%^a^) vs. mild disease (79.3%^a^) vs. incapacitating (77.7%^a^) - Comorbidities: three or more (82.2%^a^) vs. two (80.2%^a^) vs. one (79.2%^a^) vs. none (80.8%^a^)
Warren 2018 [39]	United States (*n* = 3482)	Billing codes used only by physical therapists	40.4% (4 weeks)	Not reported	Not reported

*Abbreviations*: *PT* Physiotherapy, *OR* Odds ratio, *CI* Confidence interval, *SES* Socioeconomic status, *ASA* American Society of Anaesthesiologists

^a^Calculated from data given in the publication

The time period after hospital discharge for which utilisation of PT was observed differed between all included studies and ranged from four [39] and 6 weeks [37] to three [35], six [36] and twelve [38] months. Likewise, the proportion of PT utilisation varied widely. The highest proportion of outpatient PT following TKA was observed in Australia, at 84.5% within the first 6 weeks after hospital discharge [37]. A similar proportion was reported by Smith et al. in the United Kingdom (79.0%) [38]. However, this proportion referred to the first year after discharge. The other study from the United Kingdom observed a utilisation of 48.2% within 6 months [36]. Warren et al. reported a proportion of 40.4% within 4 weeks in the United States [39]. The lowest proportion was found in the Danish study [35], which reported an outpatient utilisation of PT following TKA of 16.7% in the first 3 months after hospital discharge.

### Predictors for the use of PT

Two studies reported on the influence of sex on the use of PT following TKA (Table 3). Hamilton et al. [36] observed a higher proportion of PT utilisation in females compared to males (50.1% vs. 45.6% in the first 6 months), which was also confirmed in a multivariate logistic regression (OR: 1.26; 95% CI 1.01–1.58). Smith et al. [38] also reported a greater proportion of women using PT after hospital discharge (80.4% vs. 77.7% in the first twelve months).

Hamilton et al. was the only study reporting on the influence of age, finding that PT utilisation decreased with age (OR per year age decrease: 1.04; 95% CI 1.02–1.05) [36].

None of the studies reported frequencies regarding SES or physical function or included these variables in a regression model.

Further predictors influencing PT utilisation were reported by Andersen et al. [35] (type of hospital ward) and Smith et al. [38] (ethnicity, living arrangements, American Society of Anesthesiologists (ASA) grade, and number of comorbidities).

Hamilton et al. and Smith et al. further provided information on differences between PT users and non-PT users (e.g. regarding social deprivation (based on postcodes) or physical function (mean values of the metric Oxford Knee Score (OKS)), making it impossible to calculate PT use proportions) [36, 38], which was, however, outside the scope of this review.

### Comparison of PT utilisation in TKA versus THA

Two studies also reported on the proportion of outpatient PT following THA. Compared to TKA, PT utilisation following THA was lower in both studies, at 48.2% vs. 35.3% within the first 6 months after hospital discharge [36] and 79.0% vs. 53.0% within the first twelve months [38] (Table 3).

Hamilton et al. [36] also conducted a multivariate logistic regression model for PT use after THA. The influence of sex was higher in THA patients (OR: 1.60; 95% CI 1.27–2.03) than in TKA patients (OR: 1.26; 95% CI 1.01–1.58), while the influence of age did not differ (TKA: OR: 1.04; 95% CI 1.02–1.05 vs. THA: OR: 1.03; 95% CI 1.02–1.04). Furthermore, quality of life (measured by EQ-5D) was a predictor for PT utilisation in THA patients (OR: 1.54; CI 95% 1.08–1.20) but not in TKA patients.

## Discussion

This review aimed to systematically examine the existing literature regarding the utilisation of outpatient PT following TKA. Five studies assessing different time periods met the inclusion criteria. We found large variations in the proportions of PT use, ranging from 16.7 to 84.5%. Furthermore, two studies analysed predictors for the use of PT following TKA and found that female sex was associated with higher utilisation. In the two studies comparing to patients after THA, utilisation of PT was higher among those following TKA.

Surprisingly, only a very small number of studies is available on this question of high public health relevance. TKA is one of the most frequent surgeries in industrialised countries [8, 9], and a considerable proportion of patients undergoing TKA experience unfavourable outcomes such as chronic pain or a diminished range of motion [16]. High-quality evidence suggests that PT can be an effective treatment following TKA to help prevent those complications [19, 20]. However, for most industrialised countries, it is unclear to what extent these evidence-based recommendations are consistently implemented.

Furthermore, each of the five available studies assessed different time periods, ranging from four weeks to twelve months after hospital discharge, but none of the studies justified why the respective period was chosen. These varying time periods in which PT utilisation was studied might be explained by a focus on primary outcomes other than PT utilisation [35] or by differences in rehabilitation pathways between countries. Patients can receive various inpatient and outpatient services that might include PT at facilities such as specialised inpatient rehabilitation centres, skilled nursing home facilities, outpatient facilities, private practices or in home-based programs. In terms of inpatient rehabilitation following surgery, referral rates vary widely between countries. In the United Kingdom, inpatient rehabilitation is quite uncommon following TKA [41], and in Canada, the referral rate is also low (7.7%) [42]. The proportions of patients being discharged to an inpatient rehabilitation facility after TKA are higher in the United States. In a rapid review, a median of 26.0% was transferred to an inpatient rehabilitation facility and a further 23.8% to a skilled nursing facility [43]. Another study showed that 53.4% of TKA patients were discharged from hospital to a type of inpatient facility [44]. Even higher proportions are observed in Germany, where the acute care of TKA or THA patients is usually followed by 3 weeks of inpatient rehabilitation [45]. For example, Füssenich et al. reported that 72% of THA patients underwent inpatient rehabilitation following surgery [46].

Moreover, differences in the utilisation and intensity of outpatient rehabilitation exist between countries. For example, in Canada and the United Kingdom, the vast majority of TKA patients are transferred directly home after hospital discharge [41, 42]. In the United States, the above-mentioned rapid review showed a median of 34.1% being discharged home with supervision, but at a wide range from 0.6 to 44.2% [42]. In Germany, Füssenich et al. observed that 20% of THA patients receive outpatient rehabilitation for 3 weeks, where they have 4–6 h of therapeutic interventions per day [46]. Outpatient PT can also be used alongside or after specific rehabilitation services. For example, in the United Kingdom, where inpatient and outpatient rehabilitation are quite uncommon, the two included studies by Hamilton [36] (48.6% within 6 months) and Smith [38] (79.0% within 12 months) found a comparably high use of outpatient PT after TKA. In Australia, about 80% of patients are referred home, which also might explain the high proportion of outpatient PT (84.5% within 6 weeks) [37]. These considerable differences in policies and care pathways between countries have to be considered when interpreting our results.

Another factor reducing the comparability of studies on outpatient PT use following TKA is that definitions of PT are inconsistent across studies. Two of the five included studies did not even report any definition of PT [36, 37]. This is a crucial point as PT can include quite different interventions from land- and water-based group exercises to one-on-one treatments such as therapeutic exercise and manual therapy. Moreover, some types of PT interventions (e.g. electrotherapy or thermotherapy) also involve the use of specific tools. Besides the types of interventions, their frequency as well as their intensity might differ. In this review, only one of the included studies reported on frequency, e.g. in terms of numbers of PT sessions [38]. None of the included studies assessed PT intensity, despite the fact that treatment duration and number of repetitions might vary depending on the therapeutic goals [47]. Some studies assessing home health services utilisation might have included PT – amongst others – but not have reported on it separately. Inconsistent PT and exercise definitions were also used in reviews on other indications, including musculoskeletal [48–51] and neurological disorders [52, 53]. These inconsistencies hamper not only the comparability between studies but also the evaluation of appropriateness of care [54].

Overall, two of the five included studies analysed the influence of sex on outpatient PT utilisation following TKA [36, 38]. Both studies found higher utilisation in women than in men. This is in line with many other studies assessing PT utilisation in different populations like OA patients [55], rheumatoid arthritis patients [56], knee OA patients prior to TKA [26, 27], or even the general population [57]. Age is another parameter that is discussed as a factor influencing the utilisation of PT. As the only study in this review reporting on this factor, Hamilton et al. [36] found decreasing PT utilisation with age, in line with other studies on PT in musculoskeletal disorders [58]. However, results are inconsistent. Other studies in OA patients showed no association between younger age and PT but less frequent utilisation in the elderly (> 65) [55, 59] or no association at all [26, 60]. None of the included studies assessed functional status and SES as factors influencing PT utilisation. The current literature provides evidence of an impact of higher SES on the utilisation of PT in the general population [57] and in OA patients [59, 60] as well as an influence of lower functional status [60, 61].

Functional status might also be the reason why both included studies comparing TKA to THA patients found higher PT utilisation following TKA [36, 38]. The more complicated anatomy of the knee joint might result in a higher proportion of complications following arthroplasty [16], which is in line with numerous studies reporting inferior function and clinical outcomes for TKA patients [62–66]. For example, de Beer et al. surveyed patients who had undergone both primary unilateral TKA and THA, with a mean interval of 2.6 months between the first and second joint replacement. They found postoperatively greater pain levels, greater difficulty in ambulating, and greater difficulty performing activities of daily life after TKA, resulting in a longer period to achieve a satisfactory recovery status and a higher need for PT [66].

### Strengths and limitations

This is the first systematic review summarising the existing evidence on the proportion of outpatient PT utilisation following TKA. However, there are some limitations that have to be considered. We might have missed studies that did not focus on PT utilisation but mentioned such a proportion in the full text or were not listed in the classic medical databases. However, in order to minimise this risk, we used a comprehensive search strategy, and full texts were screened even if there was just a minimal chance of reported PT utilisation. In addition, we searched references lists of included studies and did not use any language restrictions. Some studies reported only specific treatments [44] or solely the absolute number of PT sessions [67], making it impossible to calculate the overall prevalence of PT utilisation. These studies might have assessed the proportion of PT but did not report them. Another aspect to be taken into account when interpreting our results is that the included studies are very heterogenous in terms of sample size, number of hospitals and in the way PT utilisation was assessed. Additionally, some included studies are of lower quality regarding, e.g., the conduct of data analysis with sufficient coverage of the identified sample, adequate sample size or using valid methods for the identification of PT utilisation. However, we performed quality appraisal using the JBI tool recommended for systematic reviews of studies on prevalence [32] and explained its results transparently. Furthermore, this heterogeneity is not a weakness of our approach but represents limitations of the included studies themselves. Results of this systematic review therefore provide a comprehensive overview of the evidence published worldwide on outpatient PT utilisation following TKA.

## Conclusion

In this systematic review using a broad search strategy without language restrictions, we found only five studies assessing the utilisation of outpatient PT after hospital discharge in patients with TKA, and only two of these studies also analysed predictors. The large differences in the proportions of patients using outpatient PT in any time period within the first year after TKA (ranging from 16.7 to 84.5%) might be explained by the different time periods assessed and by differences in care pathways between countries. Therefore, more studies on this relevant public health question from different countries are clearly needed; these studies should be large enough to analyse potential differences by age, sex, SES and physical function in order to provide the basis for clinical decisions and to identify potential groups that are prone to underutilisation. Furthermore, future studies ought to use uniform PT definitions or at least report their operationalisation as well as justifications for time periods assessed and should also describe frequency, intensity and types of interventions.

## Supplementary Information

**Additional file 1.** Search strategy.

## Data Availability

All data generated or analysed during this study are included in this published article.

## Abbreviations

AHRF: Area Health Resource File
ASA: American Society of Anaesthesiologists
CMS: Centres from Medicare & Medicaid Services
CI: Confidence interval
CoCoPop: Condition, Context, and Population
DRG: Diagnosis-related group
HRSA: Health Resources and Services Administration’s
JBI: Joanna Briggs Institute
LPR: National patient register
NJR: National Joint Registry for England
OA: Osteoarthritis
OKS: Oxford Knee Score
OR: Odds ratio
PRISMA: Preferred Reporting Items for Systematic Reviews and Meta-Analyses
PROMs: Patient Reported Outcome Measures
PT: Physiotherapy
ROM: Range Of motion
SES: Socioeconomic status
SSR: Health Insurance Register
THA: Total Hip Arthroplasty
TKA: Total Knee Arthroplasty
