# Supplementary material for: Utilisation of outpatient physiotherapy in patients following total knee arthroplasty – a systematic review
Source: BMC Musculoskelet Disord. 2021 Aug 18;22:711. doi: 10.1186/s12891-021-04600-2 (PMC8375073; doi:10.1186/s12891-021-04600-2)
Supplement: Supplementary file 1 — Additional file 1. Search strategy. [file 12891_2021_4600_MOESM1_ESM.docx]

# Additional material

Additional file 1: Search strategy

| PubMed | ("Arthroplasty, Replacement, Knee"(Mesh) OR (knee*(tw) AND (arthroplasty(tw) or replacement(tw))) OR TKA(tw) OR TKR(tw)) AND ("Physical Therapy Modalities"(Mesh) OR "Physical Therapy Specialty"(Mesh) OR physiotherap*(tw) OR physical therapy"(tw)) AND (pattern(tw) OR patterns(tw) OR prescription*(tw) OR prescribe*(tw) OR prescribing(tw) OR use(tw) OR utilization(tw) OR prevalence(tw) OR prevalences(tw) OR database(tw) OR rate(tw) OR rates(tw) OR administration(tw) OR session(tw) OR sessions(tw) OR provision(tw) OR frequency(tw) OR referral(tw) OR referred(tw)) |
| --- | --- |
| CINAHL | (MH arthroplasty, replacement, knee OR (knee* AND (arthroplasty or replacement) ) OR TKA OR TKR ) AND (MH physical therapy OR physiotherap* OR "physical therapy") AND (pattern OR patterns OR prescription* OR prescribe* OR prescribing OR use OR utilization OR prevalence OR prevalences OR database OR rate OR rates OR administration OR session OR sessions OR provision OR frequency OR referral OR referred ) |
| SCOPUS | ( ( TITLE-ABS-KEY ( knee* AND ( arthroplasty OR replacement ) ) ) OR ( TITLE-ABS-KEY ( tka OR tkr ) ) ) AND ( TITLE-ABS-KEY ( physiotherap* OR "physical therapy" ) ) AND ( TITLE-ABS-KEY ( pattern OR patterns OR prescription* OR prescribe* OR prescribing OR use OR utilization OR prevalence OR prevalences OR database OR rate OR rates OR administration OR session OR sessions OR provision OR frequency OR referral OR referred ) ) |
| PEDro | Abtract&Title: knee replacement, Method: Clinical Trial  Abtract&Title: knee arthroplasty, Method: Clinical Trial  Abtract&Title: TKR, Method: Clinical Trial  Abtract&Title: TKA, Method: Clinical Trial |
